# Emerging role of nanotechnology in treatment of non-alcoholic fatty liver disease (NAFLD)

**DOI:** 10.17179/excli2023-6420

**Published:** 2023-09-04

**Authors:** Atie Moghtadaie, Hamidreza Mahboobi, Somayeh Fatemizadeh, Mohammad Amjad Kamal

**Affiliations:** 1 Clinical Fellow in Gastroenterology and Hepatology, Digestive Disease Research Institute, Department of Gastroenterology and Hepatology, Shariati Hospital, Tehran University of Medical Sciences, Tehran, Iran; 2 Department of Gastroenterology and Hepatology, Shahid Beheshti University of Medical Sciences, Tehran, Iran; 3 Institutes for Systems Genetics, Frontiers Science Center for Disease-related Molecular Network, West China Hospital, Sichuan University, China; 4 King Fahd Medical Research Center, King Abdulaziz University, Jeddah 21589, Saudi Arabia; 5 Department of Pharmacy, Faculty of Allied Health Sciences, Daffodil International University, Dhaka 1207, Bangladesh; 6 Enzymoics, 7 Peterlee place, Hebersham, NSW 2770; Novel Global Community Educational Foundation, Australia

**Keywords:** non-alcoholic fatty liver disease, NAFLD, MAFLD, nanotechnology, nanoemulsions, inorganic nanoparticles, polymeric nanoparticles

## Abstract

Non-alcoholic fatty liver disease (NAFLD) is a prevailing health challenge that requires urgent innovative interventions. This review explores the role of nanotechnology as a promising potential in the treatment of NAFLD. It delineates the limitations of the current management strategies for NAFLD and highlights the new nanotechnology-based treatments including nanoemulsions, liposomes, micelles, polymeric nanoparticles, nanogels, inorganic nanoparticles, and zinc oxide nanoparticles. Despite the optimism surrounding the nanotechnological approach, the review underscores the need to address the limitations such as technical challenges, potential toxicity, and ethical considerations that impede the practical application of nanotechnology in NAFLD management. It advocates for collaborative efforts from researchers, clinicians, ethicists, and policymakers to achieve safe, effective, and equitable nanotechnology-based treatments for NAFLD.

See also Figure 1(Fig. 1).

## Introduction

Non-alcoholic fatty liver disease (NAFLD) has risen to prominence over recent decades, now recognized as the predominant chronic liver ailment affecting close to a billion individuals worldwide (Pydyn et al., 2020[120]; Younossi, 2019[173]; Younossi et al., 2016[174]). Not only does its impact reside in hepatic complications, but it also pertains to a range of extrahepatic manifestations (Adams et al., 2017[3]; Byrne and Targher, 2015[21]). However, it is worth noting that while our paper employs the terms NAFLD and non-alcoholic steatohepatitis (NASH) for consistency with prior literature, the official nomenclature has been subject to change. In 2020, a distinguished international panel introduced the term Metabolic dysfunction-associated fatty liver disease (MAFLD) as a more inclusive descriptor for liver disease tied to metabolic dysfunction (Eslam et al., 2020[34]). This term leans on positive diagnostic criteria, shifting the paradigm from exclusion-based to inclusion-based definitions. Despite this proposed reclassification, the transition from NAFLD to MAFLD has been a topic of contention. Some argue that a singular term may not suffice for a condition as multifaceted as NAFLD (Fouad et al., 2021[38]; Younossi et al., 2021[175]). Thus, while the debate continues, we have chosen to use NAFLD and NASH for clarity and alignment with existing literature.

NAFLD is a major health issue, closely linked to the rising prevalence of obesity and metabolic syndrome. As a potentially significant cause of liver transplantation in the coming years, it is crucial to explore effective treatment strategies for managing this condition. This review article aims to provide an overview of the current understanding of NAFLD, including its epidemiology, associated conditions, pathogenesis, signs and symptoms, and laboratory tests. Furthermore, we will discuss the existing management approaches, such as weight loss, pharmacologic therapies, and lifestyle modifications. Additionally, we will delve into the emerging role of nanotechnology in the treatment of NAFLD, examining various nanotechnology-based treatments, their potential benefits, and limitations in managing this condition. By exploring these aspects, we hope to offer valuable insights for clinicians and researchers in the field of gastroenterology, ultimately contributing to the development of more effective therapeutic strategies for NAFLD patients (Riazi et al., 2022[126]; Wang et al., 2023[165]).

### Definition

NAFLD is a spectrum of liver conditions characterized by the accumulation of excess fat in the liver cells (hepatocytes) in individuals who consume little or no alcohol. This excessive fat buildup is not caused by other known liver diseases, such as viral hepatitis, autoimmune liver disease, or hereditary disorders. NAFLD encompasses two primary forms: non-alcoholic fatty liver (NAFL) and non-alcoholic steatohepatitis (NASH) (Le et al., 2022[76]). NAFL is the milder form, involving simple steatosis or fat accumulation in the liver without significant inflammation or liver damage. In most cases, NAFL does not progress to severe liver complications. On the other hand, NASH is a more aggressive form, characterized by inflammation and liver cell damage (hepatocyte injury) in addition to fat accumulation. NASH can potentially lead to more severe liver complications, such as fibrosis, cirrhosis, liver failure, or hepatocellular carcinoma (de Vries et al., 2020[26]).

### Epidemiology

NAFLD has become increasingly prevalent worldwide, primarily due to the rising incidence of obesity, metabolic syndrome, and type 2 diabetes. As the most common chronic liver disorder, NAFLD affects a significant proportion of the global population (Parry and Hodson, 2020[112]). Epidemiological studies indicate that the prevalence of NAFLD varies among different regions and ethnic groups. In Western countries, the estimated prevalence ranges from 20 % to 30 % in the general adult population, while in Asian countries, the prevalence is between 15 % and 20 %. The prevalence of NAFLD is typically higher in individuals with obesity, type 2 diabetes, or metabolic syndrome, reaching up to 75 % in some studies (Noureddin et al., 2022[106]).

The incidence of NAFLD is also on the rise, reflecting the growing burden of this liver disorder. Risk factors associated with the development of NAFLD include age, gender, ethnicity, genetic predisposition, and lifestyle factors, such as poor diet and physical inactivity. Males and individuals of Hispanic descent appear to be at a higher risk of developing NAFLD (Ciardullo and Perseghin, 2022[24]; Le et al., 2022[76]). Understanding the epidemiology of NAFLD is crucial for informing public health policies, clinical management strategies, and the development of effective treatments. By identifying the populations most at risk and the contributing factors, targeted interventions can be implemented to mitigate the impact of NAFLD and improve patient outcomes.

### Associated conditions

NAFLD is often closely associated with various other medical conditions, making it essential to understand these connections for comprehensive patient management. Some of the most common conditions associated with NAFLD are presented here.

Obesity is indisputably one of the most significant risk factors associated with NAFLD. The connection between obesity and NAFLD is primarily due to the physiological processes triggered by excess body fat. When a person carries an excessive amount of body fat, it can initiate a series of metabolic changes, one of which is increased fat accumulation in the liver. This accumulation of fat can lead to liver inflammation and eventually result in NAFLD. This link between obesity and NAFLD is strongly backed by multiple scientific studies, which have revealed a consistent pattern of a higher Body Mass Index (BMI) and an increased waist circumference being associated with a higher prevalence of NAFLD. These studies shed light on the critical role obesity plays in exacerbating the risk of NAFLD (Machado and Cortez-Pinto, 2023[84]; Rojano et al., 2023[128]).

The relationship between Metabolic Syndrome and NAFLD is another critical point to consider. Metabolic Syndrome is a health issue that comprises a cluster of interrelated conditions, which include central obesity, hypertension, dyslipidemia, and insulin resistance. These conditions together significantly increase the risk of cardiovascular disease and Type 2 diabetes. NAFLD is often viewed as the hepatic manifestation of Metabolic Syndrome, which means that it can be seen as a liver-specific presentation of this systemic condition. This shared perspective arises because Metabolic Syndrome and NAFLD share many underlying pathophysiological mechanisms. Both conditions can be traced back to a set of similar metabolic irregularities, thereby establishing the parallel between them (Martinez-Urbistondo et al., 2022[87]; Radu et al., 2023[122]).

Type 2 diabetes is another condition that exhibits a strong correlation with NAFLD. The connection can be traced to the physiological relationship between insulin resistance-a primary characteristic of Type 2 diabetes-and NAFLD. Patients who have Type 2 diabetes face a higher risk of developing NAFLD, as both conditions share the feature of compromised insulin signaling. This metabolic disturbance can trigger the onset of NAFLD in individuals already diagnosed with Type 2 diabetes. On the other hand, those with NAFLD have an increased likelihood of developing Type 2 diabetes, again owing to the shared mechanism of insulin resistance (Ajmera et al., 2023[8]; Genua et al., 2022;[42] Vetrano et al., 2023[160]).

Dyslipidemia, which is characterized by abnormal blood lipid levels-specifically, elevated levels of triglycerides and low levels of high-density lipoprotein cholesterol (HDL-C), is another condition commonly observed in individuals with NAFLD. This correlation further underscores the elevated risk of cardiovascular disease in these patients. The reason for this association lies in the shared mechanisms between Dyslipidemia and NAFLD-both are driven by imbalances in lipid metabolism, which result in an overaccumulation of harmful lipids in the body, thereby causing cardiovascular disease and NAFLD respectively (Gaggini et al., 2013[40]; Krishan, 2016[69]; Rodriguez-Pasten et al., 2023[127]).

High blood pressure or hypertension is another condition that commonly coexists in patients diagnosed with NAFLD. Hypertension, by increasing the overall stress on the body's circulatory system, can potentially exacerbate liver damage in individuals with NAFLD. The presence of hypertension in these individuals is worrisome, as it can potentially intensify the risk of cardiovascular complications, resulting in a significantly worse prognosis for those affected. The concurrent presence of these two conditions calls for a more integrated approach to patient care, given their mutual potential to worsen each other's outcomes (Kasper et al., 2021[63], 2022[64]; Li et al., 2022[78]; Ye et al., 2020[171]).

The association between Polycystic Ovary Syndrome (PCOS) and NAFLD is worth mentioning. PCOS is a hormonal disorder primarily affecting women and is characterized by insulin resistance and hyperandrogenism, two metabolic disturbances that also play a significant role in the pathogenesis of NAFLD. The prevalence of NAFLD is significantly higher in women diagnosed with PCOS compared to the general female population. This correlation underscores the importance of screening for NAFLD in women with PCOS, as it could facilitate early diagnosis and management, thereby mitigating potential complications (Baranova et al., 2013[16]; Roy et al., 2022[130]; Spremovic Radenovic et al., 2022[150]).

Understanding these associated conditions is crucial for clinicians to provide comprehensive care for patients with NAFLD. By addressing the underlying risk factors and comorbidities, healthcare professionals can develop more effective and individualized treatment plans to manage NAFLD and improve patient outcomes.

### Pathogenesis

The pathogenesis of NAFLD is complex and multifactorial, involving various genetic, metabolic, and environmental factors. The development and progression of NAFLD can be explained through a "multiple-hit" hypothesis, which suggests that several parallel insults act together to cause liver injury. Some of the key factors and processes involved in the pathogenesis of NAFLD are mentioned here.

One of the most crucial factors involved in the development and progression of NAFLD is insulin resistance. Insulin resistance implies a state where the body's cells become less responsive to the hormone insulin, leading to a number of metabolic complications. A primary consequence of this resistance is impaired glucose uptake, which means that glucose isn't efficiently absorbed from the bloodstream. Furthermore, it also triggers increased lipolysis, a process that breaks down fats to release free fatty acids (FFAs). As a result, there's a surge in FFA levels in the blood. These excess FFAs are taken up by the liver and stored as triglycerides, which eventually results in hepatic steatosis or fatty liver disease. Hence, insulin resistance plays a central and pivotal role in the pathogenesis of NAFLD (Ding et al., 2021[27]; Marusic et al., 2021[90]; Palma et al., 2022[109]).

Another significant process that exacerbates NAFLD is lipotoxicity. Lipotoxicity refers to the harmful effects of excess FFAs and their toxic metabolites in the liver. These substances inflict cellular stress, induce inflammation, and cause oxidative damage, further complicating the state of the liver. It's this lipotoxicity that primarily contributes to the progression from simple steatosis, where fat accumulates in the liver, to non-alcoholic steatohepatitis (NASH). NASH represents a more advanced stage of NAFLD, distinguished by liver cell injury and inflammation. This underlines the detrimental role of lipotoxicity in the evolution of NAFLD (Bugianesi, 2008[20]; Ibrahim et al., 2011[53]; Rada et al., 2020[121]).

Inflammation is yet another key element involved in NAFLD's pathogenesis. When a person experiences obesity or insulin resistance, their adipose tissue, or body fat, reacts by releasing pro-inflammatory cytokines and adipokines. These molecules include tumor necrosis factor-alpha (TNF-α) and interleukin-6 (IL-6), which significantly exacerbate liver inflammation and injury. By doing so, they facilitate the development of NASH and fibrosis, a condition characterized by excessive accumulation of scar tissue in the liver. This shows how inflammation, stimulated by obesity and insulin resistance, can play a significant role in the progression of NAFLD (Aasadollahei et al., 2023[1]; Ajmal et al., 2014[7]; Fanaei et al., 2021[36]; Martin-Rodriguez et al., 2019[89]; Skuratovskaia et al., 2021[146]).

Oxidative stress also participates significantly in the development and exacerbation of NAFLD. Oxidative stress refers to a state of imbalance between the production of harmful reactive oxygen species (ROS) and the body's ability to neutralize them. In the context of NAFLD, lipid peroxidation and mitochondrial dysfunction cause an accumulation of ROS in the liver. This excess ROS induces oxidative stress, leading to liver cell injury, inflammation, and fibrosis. Hence, managing oxidative stress is a key aspect of mitigating the harmful effects of NAFLD (Hong et al., 2021[50]; Martin-Fernandez et al., 2022[88]; Tutunchi et al., 2023[156]).

In recent years, researchers have also identified a link between gut microbiota, the population of microorganisms living in the gut, and NAFLD. Any changes in the composition of these microorganisms, a condition known as dysbiosis, can impact the development of NAFLD. Specifically, dysbiosis can lead to an increased production of endotoxins such as lipopolysaccharides (LPS). These endotoxins can travel to the liver through the portal vein, where they trigger inflammatory and fibrotic responses. Increased gut permeability is another factor implicated in the disease's pathogenesis, facilitating the transfer of harmful substances to the liver. Thus, maintaining a healthy gut microbiota could potentially help prevent the onset of NAFLD (Kobayashi et al., 2022[66]; Oh et al., 2023[107]; Shi et al., 2021[140]).

Finally, genetic factors are crucial in determining an individual's susceptibility to NAFLD. Studies have shown that genetic predisposition and variations in specific genes can significantly influence the likelihood of an individual developing NAFLD. Certain genetic variations, particularly in the PNPLA3 and TM6SF2 genes, not only influence susceptibility but can also affect the severity of the disease. This genetic perspective adds another layer of complexity to the pathogenesis of NAFLD, underscoring the multifactorial nature of the disease (Boonvisut et al., 2017[19]; Longo et al., 2022[80]; Recuero et al., 2022[124]; Sliz et al., 2018[147]; Targher et al., 2019[152]; Villavicencio et al., 2022[161]).

Understanding the complex pathogenesis of NAFLD is crucial for the development of targeted therapeutic interventions to halt or reverse the progression of this liver disorder. By addressing the underlying mechanisms and risk factors, clinicians can better manage NAFLD and improve patient outcomes.

### Sign and symptoms

NAFLD is often a silent condition, with many individuals remaining asymptomatic, especially during the early stages of the disease. As a result, NAFLD is frequently discovered incidentally during routine blood tests or imaging studies for unrelated reasons. However, some individuals with NAFLD may experience various signs and symptoms. In this part, some of these signs and symptoms are presented.

While NAFLD can often be asymptomatic, some individuals may experience a range of signs and symptoms. For instance, a general feeling of tiredness and weakness, or fatigue, is a common yet nonspecific symptom reported by individuals with NAFLD. Though fatigue can have many different causes and can be subjective in nature, it can significantly impact the individual's quality of life and daily functioning. It is also worth noting that the presence of persistent, unexplained fatigue could be suggestive of underlying liver dysfunction and should prompt further investigation (Newton et al., 2008[103]).

Some patients with NAFLD may also report experiencing abdominal discomfort, which often manifests as mild to moderate discomfort or pain in the upper right part of the abdomen. This region corresponds to the location of the liver, and discomfort here could be indicative of liver disease. However, like fatigue, abdominal discomfort is a nonspecific symptom and could result from various conditions, hence the need for careful evaluation (Julian et al., 2022[58]; Radu et al., 2023[122]; Vetrani et al., 2022[159]).

Hepatomegaly, or an enlarged liver, is another sign that can be associated with NAFLD. This condition is typically detected during a physical examination, often as a result of fat accumulation within the liver cells. The detection of hepatomegaly could provide a valuable clue to the presence of NAFLD, given its direct connection to liver health (Abo-Amer et al., 2020[2]; Kruger et al., 2010[70]; Yap et al., 2011[170]).

In the more advanced stages of NAFLD, such as Non-Alcoholic Steatohepatitis (NASH) or cirrhosis, additional signs and symptoms may become apparent. One of these signs is jaundice, characterized by a yellowing of the skin and eyes. Jaundice results from the accumulation of bilirubin, a waste product that the liver is unable to process effectively due to advanced liver disease. The appearance of jaundice can thus suggest significant liver dysfunction (Akuta et al., 2018[10]; Hegarty et al., 2018[47]; Poornima et al., 2022[118]).

Another sign of advanced NAFLD is ascites, defined as fluid accumulation in the abdominal cavity. This condition results from increased pressure in the liver's blood vessels due to liver scarring and reduced liver function. The presence of ascites is typically indicative of a more severe stage of liver disease (Ahmed et al., 2023[5]; Reinson et al., 2023[125]).

Edema, or swelling in the legs and feet caused by fluid retention, can also occur in advanced NAFLD. This condition can develop as a result of decreased liver function and portal hypertension, a condition characterized by increased blood pressure within the liver's portal vein system. Edema can cause discomfort and mobility issues, further lowering the individual's quality of life (Yu et al., 2023[176]).

Gastrointestinal bleeding may also occur in advanced stages of NAFLD. The presence of blood in the vomit or stool may indicate bleeding from enlarged veins, known as varices, in the esophagus or stomach. This complication can develop as a result of portal hypertension and is a serious sign that requires immediate medical attention (Zuberbuhler and Boursier, 2019[178]).

Given these potential signs and symptoms, it is critical for healthcare providers to maintain a high level of vigilance in monitoring individuals with NAFLD. Early detection and intervention can significantly help prevent the progression of NAFLD and reduce the risk of serious liver-related complications. It underscores the vital role of regular medical check-ups and open patient-provider communication in managing NAFLD effectively.

### Laboratory tests

Diagnosing NAFLD involves a combination of clinical assessment, imaging studies, and laboratory tests. These tests help to identify the presence of liver injury, assess liver function, and exclude other potential causes of liver disease. Some of the most common laboratory tests used in the evaluation of NAFLD are discussed in this part.

In the evaluation of NAFLD, several laboratory tests serve as crucial tools to ascertain the condition's severity and guide subsequent treatment options. One of the most common among these tests is the Liver Function Tests (LFTs). LFTs comprise a panel of blood tests designed to measure the levels of various liver enzymes and other markers associated with liver function. These markers offer valuable insight into the health status of the liver. Particularly in patients with NAFLD, levels of specific enzymes such as alanine aminotransferase (ALT) and aspartate aminotransferase (AST) are often elevated. These elevated levels signal potential liver injury, which is characteristic of NAFLD. However, it is important to remember that normal LFT results do not necessarily exclude the presence of NAFLD. Some individuals with NAFLD may still have normal enzyme levels (Kalra et al., 2022[61]; Koot and Benninga, 2017[67]; Teo et al., 2021[153]).

Another commonly conducted test is the Complete Blood Count (CBC). A CBC provides an overview of the various types of cells present in the blood, including red blood cells, white blood cells, and platelets. This test offers vital information about the patient's overall health status and can hint at other potential health issues. By providing a comprehensive look at the body's cellular makeup, a CBC contributes to the broader picture of a patient's health, including the presence and progression of NAFLD (Niaz et al., 2011[104]).

Measurement of the lipid profile is another important laboratory test used in NAFLD evaluation. This test examines the levels of cholesterol, triglycerides, and lipoproteins in the blood. Individuals with NAFLD often have abnormal lipid levels, reflecting disruptions in their lipid metabolism. Therefore, lipid profile tests can be particularly helpful in identifying patients at risk of developing NAFLD and in providing insights into the disease's pathogenesis (Luef et al., 2009[83]; Nejati et al., 2022[99]; Wang et al., 2022[166]).

Tests for fasting blood glucose and HbA1c (glycated hemoglobin) provide an indication of the patient's glucose metabolism. These tests measure blood sugar levels over different time spans, and their results can indicate the presence of insulin resistance or type 2 diabetes. Given the close association between insulin resistance, type 2 diabetes, and NAFLD, assessing blood glucose levels is critical to identify patients at risk and guide appropriate management strategies. This makes these tests instrumental in managing NAFLD effectively (Jun et al., 2011[59]; Nejati et al., 2022[99]; Shinde and M, 2020[141]).

Viral hepatitis serology is also necessary, especially blood tests for Hepatitis B and C. These tests help rule out viral hepatitis as the cause of liver disease in patients suspected to have NAFLD. By excluding these potential causes, the clinician can be more confident in diagnosing NAFLD and formulating a treatment plan (Golabi et al., 2022[44]; Kumar et al., 2013[71]).

Autoimmune markers also play an important role in the evaluation of NAFLD. These include tests for antinuclear antibodies (ANA), anti-smooth muscle antibodies (ASMA), and liver-kidney microsomal antibodies (LKM). Such markers can help rule out autoimmune liver diseases, such as autoimmune hepatitis, which could otherwise be confused with NAFLD. Identifying these markers ensures that the diagnosis of NAFLD is as accurate as possible (Mitra and Ray, 2020[93]; Muller et al., 2016[95]; Vuppalanchi et al., 2012[164]).

Finally, iron studies, including assessing serum iron, ferritin, and transferrin saturation, form an essential part of the NAFLD evaluation. These tests help exclude hereditary hemochromatosis, a condition characterized by excessive iron accumulation that can lead to liver disease, among other complications. By ruling out this potential cause, physicians can be more confident in diagnosing NAFLD and implementing an appropriate treatment strategy.

In some cases, a liver biopsy may be performed to confirm the diagnosis of NAFLD and assess the severity of liver injury, inflammation, and fibrosis. However, due to the invasive nature of this procedure, it is generally reserved for cases where the diagnosis is uncertain or when more advanced stages of the disease, such as NASH or cirrhosis, are suspected.

## Current Management of NAFLD

### Weight loss

One of the most effective management strategies for NAFLD is weight loss. This approach has been substantiated by numerous studies that have shown how shedding excess weight can considerably ameliorate liver health and lessen the severity of NAFLD. This feat can be achieved through a mix of alterations in dietary habits and an uptick in physical activity. It is noteworthy that weight loss doesn't have to be drastic to be impactful. Even a modest reduction in weight of about 5-10 % of body weight can bring about significant improvements in liver health and the severity of NAFLD (Garcia-Compean et al., 2023[41]; Pouwels et al., 2022[119]).

When it comes to dietary modifications, the adoption of a healthier eating plan is pivotal for individuals with NAFLD striving to lose weight. Consuming a balanced diet abundant in fruits, vegetables, whole grains, and lean proteins can aid in cutting calorie intake, which is instrumental for weight loss. The Mediterranean diet is particularly recommended for patients dealing with NAFLD. This diet is characterized by high consumption of plant-based foods, healthy fats like olive oil, and a moderate intake of fish and poultry. It has been consistently proven to be beneficial for patients with NAFLD, helping to improve liver health and manage the disease effectively (Carroll and Rotman, 2023[22]; Policarpo et al., 2022[116]; Romero-Gomez et al., 2022[129]).

Regular physical activity is equally important in the journey to weight loss and overall health improvement. Exercise has multiple benefits - it can enhance insulin sensitivity, diminish inflammation, and facilitate fat loss from the liver. Activities such as walking, jogging, swimming, and cycling can significantly reduce liver fat, thereby improving NAFLD. In addition to aerobic exercises, resistance training also has an important role as it boosts muscle mass and optimizes metabolism, all contributing to weight loss and improved liver health (Henry et al., 2023[48]; Hosseini et al., 2022[51]; Kosmalski et al., 2023[68]; Wong et al., 2022[167]).

Alongside dietary alterations and increased physical activity, further lifestyle modifications, such as quitting smoking and reducing alcohol consumption, can significantly influence liver health and mitigate the risk of NAFLD progression. Smoking cessation is particularly impactful as it directly ameliorates liver health by reducing oxidative stress and inflammation, two key processes in NAFLD progression. Moreover, smoking's adverse effects extend to other NAFLD-associated comorbid conditions like cardiovascular diseases, diabetes, and metabolic syndrome, providing another compelling reason to quit. Alcohol reduction, meanwhile, holds substantial importance for patients with NAFLD. Despite NAFLD being defined by its development in the absence of heavy alcohol consumption, even moderate drinking can exacerbate liver damage, fast-tracking NAFLD's progression towards severe forms like NASH or cirrhosis. Consequently, minimizing alcohol intake or abstaining entirely can drastically lower the risk of NAFLD progression and contribute significantly to overall liver health. This comprehensive approach, incorporating both dietary and lifestyle changes, offers an effective strategy for managing NAFLD and enhancing the overall health and well-being of affected individuals (Blomdahl et al., 2023[18], Jang et al., 2023[55]).

Healthcare providers have an essential role in this journey towards weight loss and better liver health. They can provide guidance on diet and exercise, help set achievable goals, and monitor progress on a regular basis. Involving a multidisciplinary team, including dietitians, exercise specialists, and behavioral therapists, can enhance the success of weight loss interventions and provide the patient with comprehensive support to manage NAFLD effectively. This holistic approach to care ensures that all aspects of the patient's health are taken into consideration, leading to more successful outcomes in the management of NAFLD.

### Pharmacologic therapies

Apart from weight loss and lifestyle modifications, pharmacologic therapies play a significant role in the management of NAFLD. While there is currently no FDA-approved medication specifically for the treatment of NAFLD, several drugs have shown promising results in improving liver health and reducing disease progression. These pharmacologic therapies target various aspects of NAFLD pathogenesis, such as insulin resistance, inflammation, and lipid metabolism. In the following subsections, we will discuss some of the most commonly used medications in the management of NAFLD, including their mechanism of action, efficacy, and potential side effects.

### Vitamin E

Vitamin E is a fat-soluble antioxidant that has been studied for its potential benefits in the management of NAFLD. Its antioxidative properties help to protect liver cells from damage caused by oxidative stress, which is a key contributor to the pathogenesis of NAFLD. Additionally, vitamin E has been shown to improve insulin sensitivity and reduce inflammation in the liver (Du et al., 2023[30]; Ekhlasi et al., 2017[32]; Oliveira et al., 2003[108]; Panera et al., 2022[110]).

Several clinical trials have demonstrated the effectiveness of vitamin E in patients with non-alcoholic steatohepatitis (NASH), a more advanced stage of NAFLD characterized by liver inflammation and fibrosis. In these studies, vitamin E supplementation led to significant improvements in liver enzyme levels, liver histology, and markers of inflammation (Perumpail et al., 2018[113]; Poonyam et al., 2022[117]; Yoneda et al., 2015[172]).

The recommended dose of vitamin E for the treatment of NASH is typically 800 IU per day. However, it is important to note that long-term use of high-dose vitamin E may be associated with some potential risks, such as increased bleeding tendency and a higher risk of prostate cancer in men. Therefore, the decision to use vitamin E in the management of NAFLD should be based on a thorough evaluation of the patient's overall health and potential risks and benefits (Ji, 2015[57]; Podszun et al., 2020[115]).

In conclusion, vitamin E is a promising pharmacologic therapy for the management of NAFLD, particularly in patients with NASH. However, further research is needed to better understand its long-term safety and efficacy, as well as to identify the optimal dosing regimen and patient population that may benefit the most from vitamin E supplementation.

### Aspirin

The use of aspirin in managing NAFLD has attracted interest in the medical community recently. Aspirin, an over-the-counter medication known for its anti-inflammatory and anti-platelet properties, may offer additional benefits to patients with NAFLD (Sookoian and Pirola, 2017[149]).

The mechanism of aspirin's benefit in NAFLD is not completely understood, but it is theorized to stem from its ability to inhibit cyclooxygenase (COX), an enzyme involved in inflammation. By blocking COX, aspirin might reduce liver inflammation and fibrosis, key aspects of NAFLD progression (Vonghia et al., 2015[163]).

Several observational studies have suggested a protective effect of aspirin in NAFLD. A study by Lee et al. (2019[77]) indicated that aspirin users showed lower severity of liver fibrosis compared to non-users. Further, a research study by Simon et al. (2020[143]) demonstrated that long-term aspirin use is linked with reduced risk of hepatic steatosis and lower liver fibrosis scores.

Despite these encouraging findings, it is crucial to note that the beneficial effects of aspirin are mostly observed with long-term use, often greater than 5 years. Moreover, these studies are observational in nature, hence it is difficult to establish a direct causal relationship. There are currently no clinical trials providing strong evidence for the routine use of aspirin in NAFLD management (Sookoian and Pirola, 2016[148]).

Another point of consideration is the potential for side effects. Aspirin use is associated with risks, including gastrointestinal bleeding and renal insufficiency, which may outweigh its potential benefits in NAFLD. In particular, patients with advanced liver disease are at increased risk of these complications due to their existing coagulopathy and renal dysfunction (Lanas et al., 2015[74]).

In conclusion, while aspirin presents a promising potential therapeutic avenue in the management of NAFLD, further robust randomized controlled trials are needed to clarify its role. Furthermore, clinicians must consider the balance between potential benefits and risks when prescribing aspirin in this patient population. The future of aspirin in NAFLD management, thus, remains a subject of active investigation.

### Atorvastatin

Atorvastatin, a widely used medication in the statin family, holds great promise in the management of NAFLD. Statins, primarily used for controlling hypercholesterolemia, have also been found to exhibit anti-inflammatory and antioxidant properties, potentially benefiting NAFLD patients (Athyros et al., 2010[13]). The action of atorvastatin on NAFLD may be mediated through its capacity to lower serum low-density lipoprotein (LDL) cholesterol, a recognized risk factor for NAFLD. By lessening LDL levels, atorvastatin might aid in reducing hepatic lipid accumulation, a key pathophysiological feature of NAFLD (Nelson et al., 2009[100]).

Several studies have provided initial support for the use of atorvastatin in NAFLD. For instance, a randomized controlled trial by Athyros et al. (2010[13]) found improvements in liver enzymes and ultrasound findings in NAFLD patients treated with atorvastatin. A more recent study by Dongiovanni et al. (2022[28]) suggests atorvastatin may slow the progression of NAFLD to more severe stages, such as non-alcoholic steatohepatitis (NASH) or cirrhosis. However, the application of atorvastatin in NAFLD treatment is still a topic of ongoing discussion. While some evidence suggests benefits, other studies have not observed significant improvements in hepatic steatosis or fibrosis with atorvastatin use. This discrepancy might be due to differences in study design, patient population, or duration of treatment (Kargiotis et al., 2015[62]).

Further, concerns exist regarding the safety of statins in patients with liver disease, as these medications are metabolized in the liver and could theoretically exacerbate liver injury. Nonetheless, current evidence generally supports the safety of statins, including atorvastatin, in patients with chronic liver disease and NAFLD, provided appropriate monitoring is conducted (Vargas et al., 2017[158]).

In summary, atorvastatin presents as a potential therapy for NAFLD, especially for patients with co-existing dyslipidemia. However, further investigation is needed to establish definitive guidelines for its use in this context. Clinical trials examining the long-term efficacy and safety of atorvastatin in NAFLD patients are eagerly awaited.

### Omega-3 fatty acids

Omega-3 fatty acids are a form of polyunsaturated fats well known for their health benefits, including their role in heart health. There is growing interest in the potential use of omega-3 fatty acids in the treatment of NAFLD (Scorletti and Byrne, 2013[135]). The effect of omega-3 fatty acids in NAFLD may be due to their anti-inflammatory and lipid-lowering properties. In particular, they may reduce triglyceride levels, a type of fat known to accumulate in the liver in NAFLD (Parker et al., 2012[111]).

Research data on omega-3 fatty acids' effect on NAFLD is somewhat mixed. Some clinical trials show positive outcomes with supplementation. A study by Nogueira et al. (2016[105]) reported that supplementation with omega-3 fatty acids improved liver fat content and inflammatory markers in patients with NAFLD. In contrast, other studies such as a trial by Scorletti et al. (2014[134]) found no significant changes in liver fat or fibrosis scores with omega-3 supplementation.

The mixed results might be due to differences in study design, dosage, duration of therapy, and the population studied. For example, the beneficial effects might be more pronounced in those with more severe forms of NAFLD or in those with specific dietary habits or genetic profiles (Sanyal et al., 2014[132]). In terms of safety, omega-3 fatty acid supplements are generally well-tolerated. Side effects are usually mild, including digestive discomfort or unpleasant taste. Unlike some other treatments for NAFLD, they have no known risk of severe liver-related adverse effects (Parker et al., 2012[111]).

In conclusion, while omega-3 fatty acids offer an interesting potential tool in the treatment of NAFLD, more research is needed. Future studies should focus on establishing optimal dosage, duration, and identifying the patient groups most likely to benefit. Until then, the application of omega-3 fatty acids in the treatment of NAFLD remains a promising but yet to be fully validated strategy.

### Thiazolidinediones

In the therapeutic armamentarium against NAFLD, thiazolidinediones represent a class of drugs that have demonstrated promising results. These medications, also known as glitazones, are traditionally used in managing type 2 diabetes due to their insulin-sensitizing effects (Musso et al., 2012[96]). The benefit of thiazolidinediones in NAFLD could be explained by their ability to decrease insulin resistance, a key factor in the development and progression of NAFLD. Furthermore, these drugs might modulate lipid metabolism and reduce liver inflammation, which could lead to improvements in liver histology (Sanyal et al., 2010[133]).

There are two commonly used drugs in this class, pioglitazone and rosiglitazone. A seminal study by Aithal et al. (2008[6]) showed pioglitazone improved liver histology in NAFLD patients with insulin resistance . However, its use is often limited by side effects like weight gain and fluid retention, which could aggravate heart failure in susceptible individuals. Rosiglitazone has also been studied, but its use has diminished due to concerns about cardiovascular safety. In a trial by Ratziu et al. (2008[123]), rosiglitazone did not demonstrate significant improvements in liver histology compared to placebo.

Thus, while thiazolidinediones exhibit potential benefits in NAFLD, they are not currently recommended as first-line therapy due to their side-effect profile and questions about their long-term efficacy and safety. Recent guidelines suggest their use could be considered in patients with biopsy-proven NASH who are not at high risk for cardiovascular disease or heart failure (Chalasani et al., 2018[23]).

In conclusion, the role of thiazolidinediones in NAFLD management is yet not well-defined. More research is needed to determine the exact benefits, optimal duration of therapy, and which subgroups of patients may benefit the most. For now, the use of these agents should be individualized, taking into account the potential risks and benefits.

### Metformin

Metformin, a first-line medication for type 2 diabetes, has been evaluated for its potential in the management of NAFLD. Its action in NAFLD could be linked to its ability to reduce insulin resistance, a major contributor to the pathogenesis of NAFLD (Musso et al., 2010[97]). Clinical trials assessing metformin's role in NAFLD have provided mixed results. An early study by Nair et al. (2004[98]) found metformin improved liver function tests in NAFLD patients. However, subsequent randomized controlled trials, including a study by Haukeland et al. (2009[46]), found no significant difference in liver histology between metformin and placebo groups.

It is important to note that these conflicting results might be due to variations in study design, duration of treatment, or patient population. For example, metformin may be more effective in NAFLD patients with co-existing diabetes or obesity, but more studies are needed to confirm this speculation (Musso et al., 2012[96]). In terms of safety, metformin is generally well-tolerated. However, it must be used cautiously in patients with impaired kidney function due to the risk of lactic acidosis, a rare but potentially serious side effect (Inzucchi et al., 2014[54]). Given the currently available evidence, metformin is not recommended as a specific treatment for NAFLD according to the American Association for the Study of Liver Diseases. Nonetheless, its potential utility in this context should not be entirely dismissed. Metformin can still be used to manage associated conditions, such as diabetes and obesity, which are prevalent in NAFLD patients (Chalasani et al., 2018[23]).

In conclusion, while metformin holds promise, it is not currently advocated for the routine treatment of NAFLD. More large-scale, long-term trials are needed to better delineate its role in the management of NAFLD.

### Liraglutide

Liraglutide, a glucagon-like peptide-1 (GLP-1) receptor agonist, is currently approved for the management of type 2 diabetes and obesity. Its potential utility in treating NAFLD is now an area of active research (Armstrong et al., 2016[12]). The potential benefits of liraglutide in NAFLD may come from its ability to regulate glucose metabolism, promote weight loss, and possibly reduce liver fat content. The drug may also exert anti-inflammatory and anti-fibrotic effects on the liver (Marso et al., 2016[86]).

The key evidence for liraglutide's use in NAFLD comes from the LEAN trial (Armstrong et al., 2016[12]). In this randomized controlled trial, more patients in the liraglutide group achieved resolution of non-alcoholic steatohepatitis (NASH) without worsening fibrosis, compared to the placebo group (Armstrong et al., 2016[12]). While these results are encouraging, it should be noted that the trial was relatively small, with only 52 patients in the liraglutide group. Thus, larger studies are needed to validate these findings (Newsome et al., 2021[102]).

In terms of safety, liraglutide is generally well-tolerated, but side effects, including gastrointestinal symptoms such as nausea, vomiting, and diarrhea, can occur. Importantly, no major liver-related adverse effects have been reported (Pi-Sunyer et al., 2015[114]). Given the current evidence, liraglutide cannot be recommended as a routine treatment for NAFLD. However, it might be considered in patients with NAFLD who also have type 2 diabetes or obesity, conditions where liraglutide has proven benefits (Chalasani et al., 2018[23]).

In conclusion, liraglutide represents a promising potential therapeutic avenue in the management of NAFLD, but further larger-scale, long-term studies are needed to definitively establish its role. For now, the use of liraglutide in NAFLD patients should be considered on an individual basis, with careful consideration of the potential benefits and risks.

### Obeticholic acid and other FXR agonists

Obeticholic acid (OCA) has emerged as a significant player in the arena of NAFLD and NASH treatments due to its role as a farnesoid X receptor (FXR) agonist. The critical function of FXR in overseeing bile acid synthesis, transport, and absorption has been well established. As a consequence, when OCA interacts and activates this receptor, it has the potential to steer lipid metabolism in a direction that curbs inflammation in the liver. This modulation can act as a brake, potentially decelerating or even reversing the menacing progression witnessed in NAFLD and NASH affected patients (Liu et al., 2023[79]).

Moreover, clinical trials focusing on OCA have yielded encouraging results, particularly in the realm of reducing liver fibrosis without exacerbating inflammation. However, like all therapeutic candidates, OCA is not devoid of challenges. Clinicians and patients need to be wary of potential side effects. Notably, issues like pruritus and a spike in LDL cholesterol levels have been observed. Such side effects underscore the importance of rigorous monitoring when this treatment is administered (Neuschwander-Tetri et al., 2015[101]).

### Nor-UDCA

In the ever-evolving landscape of NAFLD/NASH treatments, nor-ursodeoxycholic acid (nor-UDCA) has carved a niche for itself, being a distinct bile acid derivative. What differentiates nor-UDCA from the standard UDCA is its behavior in the liver; it refrains from conjugation, thereby enhancing its cholehepatic shunting prowess. This unique mechanism of action encourages ductular reactions and stimulates bicarbonate secretion, acting as a shield against the ravages of bile acid-induced damage to cholangiocytes (Jansen, 2018[56]).

Early-stage clinical trials revolving around nor-UDCA have painted an optimistic picture. The compound has demonstrated its capability to considerably diminish liver enzyme levels. Such a decline is often interpreted as a signal indicating a reduction in liver inflammation and the associated damage, hallmarks of NAFLD/NASH conditions (Fickert et al., 2017[37]).

### Anti-inflammatory agents

Given the deeply ingrained inflammatory profile of NASH, it is logical to earmark anti-inflammatory agents as potential therapeutic warriors against this ailment. These agents, with their diverse mechanisms of action, aim their sights on different nodes of the expansive inflammatory cascade. Their ultimate goal is to dampen the inflammatory response, thereby reducing the chances of hepatocellular injury and the dreaded specter of subsequent fibrosis (Loomba and Sanyal, 2013[81]). 

One such noteworthy agent in this category is cenicriviroc, which has been designed to target the CCR2 and CCR5 receptors. These receptors play a crucial role in the recruitment and activation of macrophages. The clinical trials involving cenicriviroc have produced a spectrum of results. While not universally positive, there is a growing belief within the scientific community that by finetuning aspects like dosing and ensuring targeted delivery, the full potential of these anti-inflammatory agents can be harnessed. There's also a line of thought suggesting that the real game-changer might be a combination therapy, merging the strengths of anti-inflammatory agents with other therapeutic avenues (Friedman et al., 2018[39]).

## New Nanotechnology-Based Treatments

Nanotechnology, the manipulation of matter on a molecular and atomic level, is an exciting frontier in medical research. Its application in NAFLD treatment offers hope for improved therapeutic strategies.

### Nanoemulsions

In the field of nanotechnology, nanoemulsions show significant potential for the treatment of NAFLD. These are systems consisting of oil and water, mixed into one phase by means of surfactants, and have the characteristic of maintaining stability for a long time. With the size in nanoscale range, these emulsions have high interfacial area that is beneficial for drug encapsulation and release (Ahmad et al., 2017[4]; McClements, 2012[92]).

In the context of NAFLD, nanoemulsions can be used for targeted delivery of therapeutic agents to the liver. Encapsulating the drug in the nanoemulsion allows for improved bioavailability and can decrease the effective dosage of the drug, reducing potential side effects (Elbaset et al., 2022[33]; Tötterman et al., 2004[155]). Promisingly, studies have shown that using nanoemulsions can enhance the effectiveness of therapeutic agents in the management of NAFLD. For instance, curcumin, a natural polyphenolic compound with potent antioxidant and anti-inflammatory properties, has poor bioavailability due to its rapid metabolism and systemic elimination. However, curcumin-loaded nanoemulsions have shown an enhanced therapeutic effect in NAFLD by improving its bioavailability (Yallapu et al., 2015[169]).

In summary, nanoemulsions represent a promising nanotechnology-based strategy for enhancing the delivery of therapeutic agents in the treatment of NAFLD. More research is necessary to optimize these formulations and evaluate their long-term safety and efficacy in patients.

### Liposomes

Liposomes are another type of nanostructured delivery system, displaying valuable potential in the management of NAFLD. A liposome is a spherical vesicle composed of at least one lipid bilayer. These nanostructures have the capability to encapsulate both hydrophilic and hydrophobic drugs, resulting in improved solubility, enhanced bioavailability, and decreased toxicity of the drugs (Ghasemzadeh et al., 2018[43]; Sercombe et al., 2015[136]). Specific to NAFLD, liposomes can deliver therapeutic compounds to the liver in a targeted way. Because of their size and structure, liposomes can be taken up by the liver's sinusoidal endothelial cells, making them an efficient way of delivering drugs to hepatic tissues (Avachat and Patel, 2015[14]).

Liposomes can serve as effective carriers for a range of potential therapeutic agents for NAFLD. For example, the compound silybin, a component of milk thistle, has shown potential for NAFLD treatment. However, its poor solubility and low bioavailability limit its use. The encapsulation of silybin in liposomes has been studied and results show an increased bioavailability and liver targeting, improving its effectiveness in the management of NAFLD (Kidd and Head, 2005[65]).

However, while the potential of liposomes in NAFLD treatment is substantial, further research is needed to optimize their properties for enhanced delivery and to study their long-term safety and efficacy in patients with NAFLD. The development of the liposome technology holds significant promise in advancing the treatment of NAFLD and should be further explored.

### Micelles

In the arena of nanotechnology, micelles represent a promising system for drug delivery, particularly in the context of NAFLD. Micelles are self-assembling nanosized particles, typically formed from amphiphilic molecules. These structures have a unique ability to encapsulate hydrophobic drugs in their core, allowing for improved solubility and bioavailability (Kumari et al., 2014[72]; Lu and Park, 2013[82]). In terms of NAFLD management, micelles offer a strategy for targeted drug delivery to hepatic tissue. The small size of micelles, usually around 10-100 nm, permits them to effectively bypass biological barriers, enhancing their delivery potential to specific cells or tissues (Torchilin, 2014[154]).

One good example of the application of micelles is in the delivery of curcumin, a natural polyphenolic compound that exhibits significant anti-inflammatory and antioxidant properties, making it potentially beneficial in the treatment of NAFLD. Nevertheless, the clinical application of curcumin is limited due to its poor solubility and rapid metabolism. Curcumin-loaded micelles have been shown to enhance the solubility and improve the bioavailability of curcumin, thus improving its therapeutic effectiveness (Yallapu et al., 2012[168]).

Another advantage of micellar systems is their capacity to provide a controlled release of drugs, ensuring the maintained presence of the therapeutic agent in the body over an extended period. This feature can significantly enhance treatment effectiveness and patient compliance (Bae and Kataoka, 2009[15]).

Despite the encouraging potential of micelles in the treatment of NAFLD, there remain challenges and limitations. Stability is a major issue with micellar systems as they can be prone to disintegration in biological fluids, leading to premature drug release. Moreover, the long-term safety of micelles is yet to be fully determined. Further studies are required to address these concerns and to optimize the properties of micelles for their successful use in NAFLD treatment.

### Polymeric nanoparticles

Polymeric nanoparticles have lately been showing much promise as drug delivery systems in the treatment of NAFLD. These nanoparticles, usually ranging from 10 to 1000 nm in size, are made up of various biodegradable polymers that encapsulate therapeutic agents, providing a protective environment to enhance their stability and bioavailability (Amaral et al., 2021[11]; Kumari et al., 2010[73]; Morales-Becerril et al., 2022[94]). One significant advantage of polymeric nanoparticles is their large surface area to volume ratio, which can be modified to improve drug loading efficiency. The surface of these nanoparticles can be functionalized with specific ligands or antibodies to target diseased cells or tissues, an attribute of paramount importance in NAFLD where hepatic cells are primary targets (Danhier et al., 2012[25]).

As with other nanoparticle systems, polymeric nanoparticles offer the potential for controlled and sustained release of therapeutic agents, ensuring prolonged drug action. This is a critical factor in the management of chronic diseases like NAFLD (Makadia and Siegel, 2011[85]). Recent studies have demonstrated the efficiency of polymeric nanoparticles in delivering curcumin to hepatic cells in a NAFLD model. The encapsulation of curcumin in these nanoparticles resulted in increased bioavailability and a more pronounced therapeutic effect compared to free curcumin (Rozanski et al., 2023[131]; Wang et al., 2023[165]; Yallapu et al., 2012[168]; Zhang et al., 2023[177]).

However, potential issues of polymeric nanoparticles should not be overlooked. Although many polymers used are biodegradable and considered safe, concerns have been raised about potential toxicity, especially with long-term usage. Also, their larger size, compared to micelles or liposomes, may present challenges in terms of cellular uptake and tissue penetration. Finally, the production of polymeric nanoparticles often involves complex processes that may not be suitable for scaling up for industrial production (Hua et al., 2018[52]).

In summary, polymeric nanoparticles, with their ability to increase drug stability and enable targeted delivery, are promising candidates for NAFLD treatment. However, more research is necessary to optimize their properties, to address potential safety concerns, and to refine production processes for commercial viability.

### Nanogels

Nanogels are three-dimensional, hydrophilic polymeric networks with nanoparticle-sized structures, which have been emerging as valuable tools in drug delivery for NAFLD treatment. Their network structure, composed by cross-linked polymer chains, provides unique advantages such as high loading capacity and controlled release of incorporated drugs, leading to prolonged therapeutic effects (Hamidi et al., 2008[45]). Advances in nanogel research have allowed for their design to be tailored for desired characteristics. It is possible to modify the properties, such as size, shape, and surface functionality, to increase their stability in the body and to enhance their ability to target specific cells or tissues (Mauri et al., 2021[91]).

Nanogels have a large water content, which gives them a high degree of flexibility and biocompatibility. The porous structure of nanogels is very suitable for encapsulating hydrophilic and hydrophobic drugs, proteins, and even genetic material, all of which can be potentially beneficial in NAFLD treatment (Vinogradov et al., 2002[162]). A notable application of nanogels in NAFLD has been the encapsulation of anti-inflammatory agents, aiming to reduce inflammation in the liver. Nanogels have shown the potential to deliver these agents directly to the hepatic cells, increasing their efficiency and reducing side effects (Bhattarai et al., 2010[17]).

Moreover, nanogels can also protect the incorporated drugs from premature degradation and elimination, thus improving their bioavailability and therapeutic efficiency. Additionally, the swelling and shrinking properties of nanogels in response to changes in environmental factors such as temperature or pH can be utilized for targeted and controlled drug release (Hoare and Kohane, 2008[49]). However, potential drawbacks of nanogels should not be ignored. Issues related to possible immunogenicity, potential cytotoxicity of the polymers used, and challenges in scaling up the production processes need to be addressed. Long-term studies are necessary to determine the safety of nanogels for clinical applications (Kabanov and Vinogradov, 2009[60]).

In conclusion, nanogels with their unique physicochemical properties provide an exciting platform for drug delivery in NAFLD. The potential of nanogels in increasing therapeutic efficiency and reducing side effects positions them as promising candidates in the management of NAFLD. However, more comprehensive studies are needed to fully exploit their potential and to address the challenges.

### Inorganic nanoparticles

In the array of nanotechnology-based treatments for NAFLD, inorganic nanoparticles have also been exhibiting interesting potential. These particles are typically composed of metals, metal oxides, or ceramics, and provide several benefits for medical applications due to their unique physical and chemical properties (Dreaden et al., 2012[29]). Inorganic nanoparticles can exhibit high stability, tunable size, shape, and surface properties, which can be exploited to optimize their interactions with biological systems. Because of their small size, these particles can cross biological barriers and reach targeted cells or tissues, which can result in an increased efficiency of the carried drug (Singh et al., 2018[144]).

Gold nanoparticles (AuNPs), for instance, are one of the most studied inorganic nanoparticles. They show good biocompatibility, and their surface can be easily modified to encapsulate therapeutic agents or to attach specific ligands, allowing for targeted drug delivery. For NAFLD, AuNPs have been utilized to deliver anti-inflammatory agents or antioxidants, aiming to reduce inflammation and oxidative stress in the liver (Dykman and Khlebtsov, 2011[31]). On the other hand, magnetic iron oxide nanoparticles (IONPs) have shown promise for theranostic applications - both diagnosis and treatment of NAFLD. The magnetic properties of IONPs enable them to be used in magnetic resonance imaging (MRI) to diagnose NAFLD. In addition, IONPs can also be used to deliver therapeutic agents or to induce hyperthermia in targeted tissues for therapeutic purposes (Laurent et al., 2008[75]).

Silica nanoparticles (SiNPs) are another type of inorganic nanoparticles that are commonly used due to their good biocompatibility and the possibility of surface modification. In the context of NAFLD, SiNPs can be used to deliver drugs, and their porous structure allows for a high loading capacity and controlled release of the encapsulated drug (Tang et al., 2012[151]). Despite the mentioned promising features, the application of inorganic nanoparticles in NAFLD treatment faces challenges. These include potential toxicity, the need for appropriate surface modification to avoid rapid clearance by the immune system, and the need for targeted delivery to minimize side effects. It is also necessary to address issues related to large scale production and the reproducibility of nanoparticle synthesis (Fadeel and Garcia-Bennett, 2010[35]).

In conclusion, inorganic nanoparticles represent a promising approach in the nanotechnology-based treatments for NAFLD. Further research and optimization are needed to overcome the challenges and to fully exploit their potential.

### Zinc oxide nanoparticles

Zinc oxide nanoparticles (ZnO NPs), with their unique properties, have emerged as a promising nanotechnology-based strategy in the management of NAFLD. ZnO NPs have small size, and high surface area to volume ratio, which makes them able to penetrate cells and tissues efficiently. Also, they possess anti-inflammatory and antioxidant capacities, which are desirable attributes in the context of NAFLD, where inflammation and oxidative stress are implicated in the pathogenesis (Sharma et al., 2020[137], 2012[138]; Siddiquah et al., 2018[142]).

One interesting application of ZnO NPs in NAFLD treatment is their antioxidant capability. As NAFLD is characterized by oxidative stress, the role of ZnO NPs to quench reactive oxygen species (ROS) has been explored. Preclinical studies have shown that ZnO NPs could significantly decrease oxidative stress markers and inflammation in liver tissue, indicating their potential for NAFLD treatment (Akhtar et al., 2012[9]).

Moreover, ZnO NPs have been employed as drug carriers for NAFLD treatment. The high surface area allows ZnO NPs to load a large amount of therapeutic molecules. When conjugated with specific liver targeting ligands, ZnO NPs can efficiently deliver the loaded drugs to liver cells, thereby enhancing therapeutic efficacy while reducing systemic side effects (Sirelkhatim et al., 2015[145]). It is also worth noting that the safety of ZnO NPs has been widely investigated. While there is concern about their potential toxicity, especially when they are inhaled or applied to the skin, the oral administration of ZnO NPs has generally been considered safe, as the particles are primarily excreted in the feces and little is absorbed into the systemic circulation (Vandebriel and De Jong, 2012[157]).

However, there still exist certain challenges that need to be addressed before ZnO NPs can be successfully translated into clinical practice. The main challenges include understanding their exact mechanism of action, optimizing the dosage to maximize efficacy while minimizing potential toxicity, and standardizing the synthesis procedure for reproducibility and large-scale production (Sharma et al., 2012[139]).

In conclusion, ZnO NPs have shown promise in the treatment of NAFLD due to their antioxidant properties and their potential for targeted drug delivery. However, further investigations are needed to fully exploit their potential and to overcome the existing challenges. 

In an effort to synthesize the information discussed and offer a clear visual comparison, the key attributes of each nanotechnology-based treatment have been compiled into Table 1(Tab. 1). This table summarizes the primary benefits and drawbacks associated with each type of nanotechnology, including nanoemulsions, liposomes, micelles, polymeric nanoparticles, nanogels, inorganic nanoparticles, and zinc oxide nanoparticles. Further, it illustrates the potential use of each type in the context of NAFLD treatment. This overview allows for a simplified, side-by-side comparison to better appreciate the potential, as well as the challenges, of these diverse nanotechnologies in the management of NAFLD.

## Limitations of Nanotechnology in Management of NAFLD

As promising as nanotechnology appears to be in the realm of NAFLD treatment, it is essential to take note of the limitations and challenges it faces. These are encompassing technical aspects, toxicity, and ethical considerations that may hamper full-scale implementation.

First, we face the technical challenge. The large-scale production of nanomaterials, while maintaining uniformity in size, shape, and other physicochemical properties, is quite challenging. Additionally, maintaining the stability of nanomaterials in biological systems can be an uphill task due to potential agglomeration and changes in their properties over time.

Second, there is the issue of potential toxicity. Even though nanomaterials have shown good biocompatibility in most studies, the concern over their long-term safety still exists. Potential toxicity to the liver and other organs cannot be overlooked, as nanomaterials can interact with cellular components in unpredictable ways. The fact that they can cross biological barriers and accumulate in organs raises concerns about chronic toxicity. Furthermore, the impact of nanomaterials on the immune system, both innate and adaptive, needs to be further explored.

Third, targeted drug delivery, one of the key advantages of nanotechnology, also presents a challenge. Targeting nanomaterials specifically to liver cells affected by NAFLD is not an easy task. While ligands can be added to the surface of nanomaterials to enhance liver targeting, ensuring that they reach only the diseased cells and not the healthy ones is difficult. Moreover, controlling the release of drugs from nanocarriers at the target site for optimal therapeutic effect is another complex issue.

Moreover, there is a lack of standard guidelines and protocols for the assessment of nanomaterials' safety and efficacy in the context of NAFLD. This makes it difficult to compare the results of different studies and draw definitive conclusions. The establishment of such guidelines would be a crucial step towards the clinical translation of nanotechnology-based treatments for NAFLD.

Finally, there are also ethical considerations. The clinical use of nanotechnology raises ethical questions about patient consent, especially given the potential long-term health impacts that are still not fully understood. Additionally, the cost of nanotechnology-based treatments might be high, which could limit their accessibility to patients from low- and middle-income countries.

In conclusion, while nanotechnology holds great promise in the management of NAFLD, several limitations and challenges need to be addressed. Ongoing research efforts are required to fully realize its potential, and multidisciplinary collaboration involving scientists, clinicians, ethicists, and policymakers is crucial to ensure the safe and effective application of nanotechnology in NAFLD treatment.

## Conclusion

In the context of increasing prevalence and complexities of NAFLD worldwide, the seeking of novel therapeutic strategies has become more pressing. A clear understanding is required of the fact that nanotechnology, a promising new horizon in the field of medicine, offers a fresh hope for this daunting task. Nanotechnology's potential for targeted drug delivery and reduced systemic toxicity has made it an attractive tool for managing NAFLD. Various nanoformulations, including nanoemulsions, liposomes, micelles, polymeric nanoparticles, nanogels, inorganic nanoparticles, and zinc oxide nanoparticles, have shown promising results in preclinical studies for the treatment of NAFLD.

It is the unique properties of nanoparticles that allows them to interact with biological systems at a molecular level, potentially leading to significant advancements in the diagnosis and treatment of NAFLD. But alongside these bright potentials, it is important to not ignore the challenges that nanotechnology faces. The limitations such as technical challenges, potential toxicity, problems with targeted drug delivery, absence of standard guidelines, and ethical considerations have to be overcome before the full potential of nanotechnology in NAFLD management can be realized. Additionally, the high cost of nanotechnology-based treatments might be a hurdle in their widespread use, especially in the resource-poor settings. Therefore, it becomes vital that while we forge ahead in this direction, researchers, clinicians, ethicists, and policymakers need to work together. Such collaboration can ensure the development and implementation of safe, effective, and equitable nanotechnology-based treatments for NAFLD.

To summarize, while current traditional management strategies for NAFLD, including lifestyle modifications and existing pharmacologic therapies, continue to play a significant role, the future of NAFLD management may well be influenced by the advent of nanotechnology. The journey is far from over, but the advancements in the field of nanotechnology provide a beacon of hope in our fight against this globally significant disease. It is through continued research and collaboration that we can fully utilize the potential of nanotechnology for the benefit of NAFLD patients worldwide.

## Notes

Hamidreza Mahboobi and Somayeh Fatemizadeh (Department of Gastroenterology and Hepatology, Shahid Beheshti University of Medical Sciences, Tehran, Iran; E-mail: sfatemi19@gmail.com) contributed equally as corresponding author.

## Conflict of interest

The authors declare that there are no conflicts of interest.

## Figures and Tables

**Table 1:**
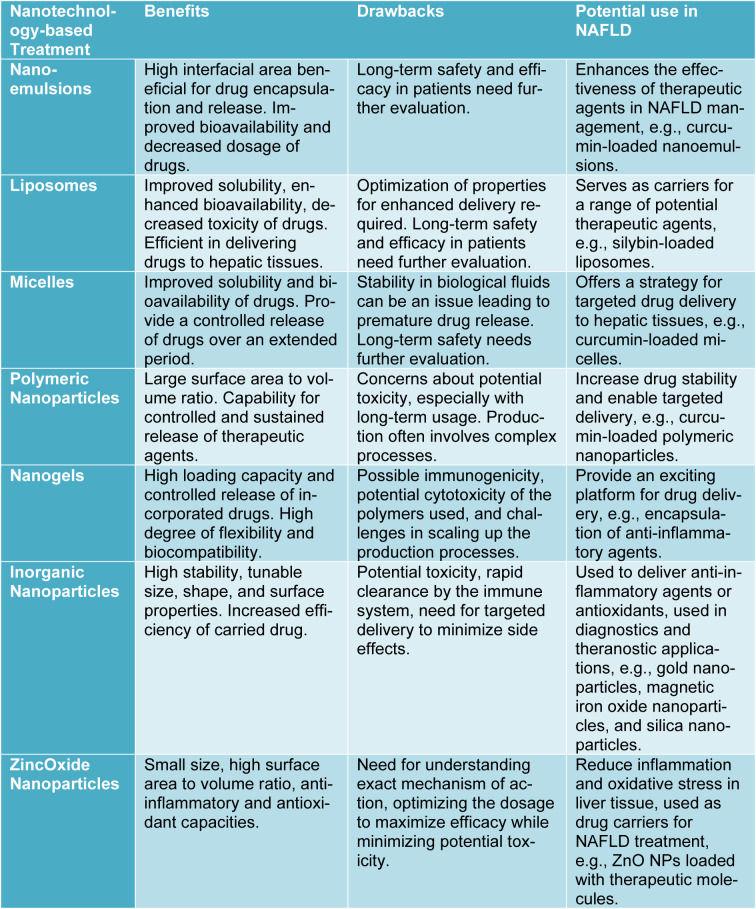
Comparative summary of nanotechnology-based treatments for Non-alcoholic Fatty Liver Disease (NAFLD).

**Figure 1:**
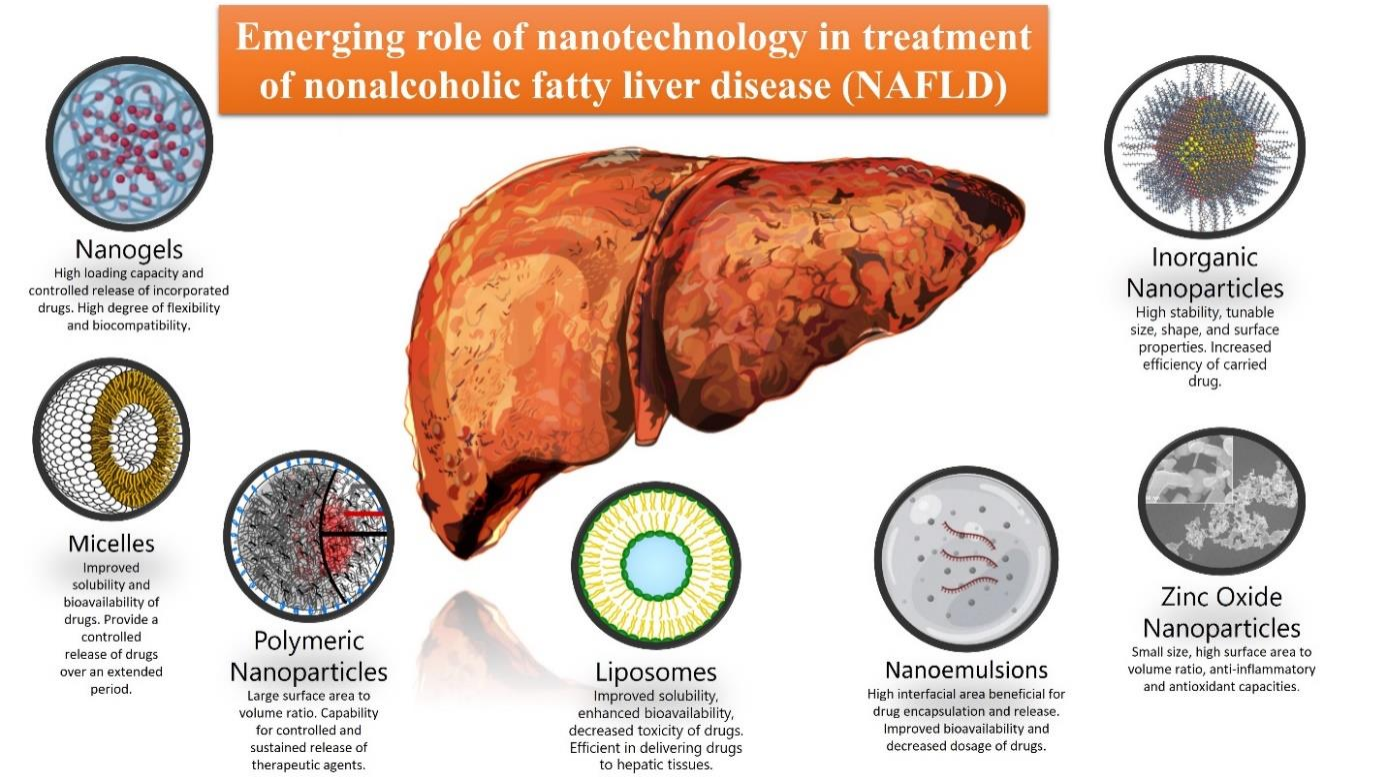
Graphical abstract - This schematic illustration provides a visual summary of the diverse nanotechnology-based strategies utilized in the treatment of Non-Alcoholic Fatty Liver Disease (NAFLD). It shows the disease condition represented by a NAFLD-affected liver, and the interaction of different nanoparticles with the hepatic cells. The nanoparticles include nanoemulsions, liposomes, micelles, polymeric nanoparticles, nanogels, inorganic nanoparticles, and zinc oxide nanoparticles, each annotated with their unique features. The illustration also underscores the key advantages of these treatments, including targeted delivery, improved bioavailability, and decreased toxicity. The graphical abstract provides a snapshot of the article's content, summarizing the key points in a concise and visually engaging manner.
